# Determinants of Vitamin D deficiency among type 2 diabetes mellitus patients: A retrospective study

**DOI:** 10.1097/MD.0000000000037291

**Published:** 2024-02-23

**Authors:** Sami Hamdan Alzahrani, Mukhtiar Baig, Khaled A. Yaghmour, Sarah Al Muammar

**Affiliations:** a Department of Family Medicine, Faculty of Medicine, King Abdulaziz University, Jeddah, Saudi Arabia; b Health Promotion Research Group, Deanship of Scientific Research, King Abdulaziz University, Jeddah; c Department of Clinical Biochemistry, Faculty of Medicine, Rabigh, King Abdulaziz University, Jeddah, Saudi Arabia.

**Keywords:** diabetes mellitus, HbA1c, impaired HbA1c, obesity, Vitamin D deficiency

## Abstract

Recent studies have shown an association between vitamin D deficiency (VDD) and type 2 diabetes mellitus patients (T2DM), but the precise relationship between these conditions has yet to be clarified. In this study, we aimed to estimate the incidence of VDD among diabetic patients and identify any relationship between diabetes and the determinants of VDD among T2DM individuals. A retrospective cross-sectional study was conducted at King Abdulaziz University Hospital, Jeddah, Saudi Arabia. Patients of either gender were selected from electronic records and checked for vitamin D levels, glycosylated hemoglobin (HbA1c), and other parameters. A total of 864 subjects were enrolled. Subjects were grouped according to HbA1c levels, with < 5.7%, 5.7% to 6.49%, and > 6.5% considered normal, impaired, and diabetic, respectively. VDD was common, with an incidence of 723 (83.7%) subjects. A significantly lower vitamin D level was found in diabetic subjects than in nondiabetic subjects (29.1 ± 12.0 vs 44.0 ± 28.3, *P* < .001). A total of 207/209 (99%) subjects with impaired HbA1c and 179/183 (97.8%) people with diabetes had VDD. Interestingly, none of the diabetic or impaired HbA1c subjects had normal vitamin D levels. A significant association was found between VDD and being > 50 years old, overweight, or obese, as well as HbA1c, fasting plasma glucose, calcium, and total cholesterol (TC) levels. A high rate of VDD and significantly lower vitamin D levels were found in diabetic subjects. Age, being overweight, obesity, HbA1c, and fasting plasma glucose were the few determinants of VDD among T2DM patients. These findings highlight the importance of addressing vitamin D status in managing and preventing T2DM, particularly in those over the age of 50, those who have higher body weight, and those with raised HbA1c and fasting plasma glucose levels.

## 1. Introduction

The World Health Organization predicts that the number of people with diabetes could reach as high as 299 million by the year 2025. It is also predicted to become the leading cause of mortality by the year 2035.^[1]^ Vitamin D deficiency (VDD) is also fairly prevalent, contributing to multiple disorders such as rickets, osteomalacia, osteoporosis, and male infertility.^[2,3]^ VDD is a worldwide concern that impacts individuals of all age groups, regardless of gender. Additionally, vitamin D has the potential to enhance insulin sensitivity by promoting greater responsiveness of insulin in facilitating glucose transport, thus reducing the risk of developing DM.^[4]^

Despite innovations in managing diabetes, mortality and morbidity have remained high. Explorations of vitamin D supplementation for the prevention of diabetes have been conducted but without satisfactory follow-up and evaluation. The epidemiology of VDD and DM are interlinked, and those with diabetes among some ethnic groups are more prone to VDD. In addition, diabetes is more commonly seen in elders, who also have an increased chance of having VDD. A study reported that vitamin D and calcium homeostasis might play an important role in the development of diabetes.^[5]^

VDD, which affects multiple tissues in addition to bone, is a major health issue in some parts of the world. Hypovitaminosis D is defined as having serum 25[OH]D levels below 50 nmol/L. A study has reported a worldwide VDD prevalence of between 50% and 80%, depending on the area of the world.^[6]^

Low levels of vitamin D are linked with an increased risk of developing resistance to insulin, metabolic syndrome, and DM. Vitamin D deficiency increases with a worsening of resistance to insulin and dysfunctional beta cells.^[7]^ An inverse correlation between vitamin D level and diabetes has also been recorded, and such a report results in hypothesizing that VDD might play a vital part in diabetes pathogenesis.^[8]^

Obesity has been associated with a higher risk of acquiring DM. It is also linked with low vitamin D levels, possibly because of decreased exposure to sunlight, increased vitamin D catabolism, or even vitamin D sequestration within the subcutaneous adipose tissues.^[9]^ Individuals with type 2 diabetes mellitus (T2DM) frequently experience multiple metabolic abnormalities that contribute to increased rates of illness and death.^[10]^ Despite the recognized importance and the extensive research that has already been conducted in the field of VD, various scientific questions and issues related to VD still demand further investigation and continued scholarly contributions.^[11]^ Diabetes prevention relies on a foundation of knowledge, a positive attitude, and a proactive dedication to adopting healthy behaviors. Diabetes education that addresses risk factors is likely to be more successful in this regard.^[12]^

While lower levels of vitamin D in diabetic patients with or without insulin resistance have been implicated in various studies, a large number of diabetic patients who have resistance to insulin are not obese. Furthermore, resistance to insulin is reported to be higher among South Asians than those from other parts of the world.^[13]^ The prevalence of diabetes in Saudi Arabians is reported to be around 30%, one of the highest rates in the world. Importantly, VDD has been associated with DM. It has been noted that resistance to insulin depends on vitamin D levels, showing a positive correlation between insulin sensitivity and levels of vitamin D. Overall, VDD is reported in 70–90% of individuals with diabetes,^[14]^ but only a handful of studies have been done in Saudi Arabia in this regard. The objectives of this study were to estimate the incidence of VDD among diabetic individuals and to determine the relationship between diabetes and VDD, as well as the determinants of VDD among T2DM individuals.

## 2. Methods

### 2.1. Study design

This retrospective cross-sectional study was carried out at King Abdulaziz University Hospital (KAUH) in Jeddah, Saudi Arabia, between December 2019 and May 2020. The protocol of the present study was approved by the Research Ethics Committee of King Abdulaziz University, Jeddah, Saudi Arabia (Reference No. 20215). Patients were selected from electronic records at the KAUH family medicine clinics. This study adhered to all applicable regulations and legislation, as well as the Helsinki Declaration. The patients’ confidentiality was maintained, and informed consent was sought from all patients via their available contact numbers. Subjects of either gender were included in the study and checked for vitamin D levels, HbA1c, fasting plasma glucose (FPG), random plasma glucose, and several other routinely measured parameters. Pregnant females, females who developed gestational diabetes, and type 1 diabetic patients were excluded from the study.

### 2.2. Data collection tool (instrument) and technique

A data collection sheet was designed in the family medicine and diabetology clinics at KAUH, and the data were collected from the electronic records of patients who had normal and impaired HbA1c or FPG readings. First, sociodemographic characteristics, including age, gender, nationality, educational level, lifestyle and exercise, type of job, if any, and family income, were recorded. Then, a history of known illnesses such as endocrine diseases (DM, dyslipidemia, hypertension, obesity) was noted, and cardiogenic disorders and any family history of medical illnesses were included. Also noted were medications the subject was taking, such as oral hypoglycemic tablets, vitamin D supplements, and other vitamins or minerals considered multivitamin supplements. The missing data were obtained from patients during subsequent visits or by contacting them individually. Anthropometric measurements, including height and weight, were also recorded, and body mass index (BMI) was calculated. The study subjects were then divided into 3 groups according to their HbA1c levels, with HbA1c < 5.7 in the normal group, HbA1c of 5.7 to 6.49 in the impaired group, and HbA1c > 6.5 in the diabetic group.^[1]^ Further grouping was done according to vitamin D levels: normal (76–250 nmol/l), insufficient (50–75 nmol/l), and deficient (<50 nmol/l).^[15]^

### 2.3. Data collection and analysis

The data were analyzed using Statistical Package for the Social Sciences 20 (SPSS Inc., Chicago, IL). The qualitative data were expressed as frequencies and percentages, while quantitative data were expressed as means and standard deviations. The Chi-square test compared basic characteristics according to vitamin D status. The Pearson correlation coefficient analysis was used to explore the correlation of vitamin D with basic characteristics among the 3 groups. Univariate and multivariate logistic regression analyses were employed to determine the association between VDD and age, overweight, obesity, HbA1c, FPG, and other variables. The level of significance was set at < 0.05.

## 3. Results

A total of 864 subjects were enrolled in the study, of which 457 (52.9%) were younger than 50 years old and 690 (79.9%) were females. Other values are shown in Table 1. There were 472 (54.6%), 209 (24.2%), and 183 (21.2%) subjects nondiabetic, impaired, and diabetic. Surprisingly, most study subjects, 723 (83.7%), had VDD (Table 1).

**Table 1:** Study subjects’ basic characteristics.

Variables	N (%)
Age	
<50 yr	457 (52.9)
>50 yr	407 (47.1)
Gender	
Female	690 (79.9)
Male	174 (20.1)
Smoking	
Yes	45 (5.2)
No	819 (94.8)
Education	
College or above	308 (35.6)
High school	103 (11.9)
Primary school	453 (52.4)
Nationality	
Saudi	501 (58)
Non-Saudi	363 (42)
Family history of DM	
Yes	144 (16.7)
No	720 (83.3)
BMI	
Underweight	32 (3.7)
Normal	210 (24.3)
Overweight	243 (28.1)
Obese	379 (43.9)
Vitamin D	
Normal	77 (8.9)
Insufficient	64 (7.4)
Deficient	723 (83.7)
HbA1c	
Normal (Control; HbA1c < 5.7)	472 (54.6)
Impaired (HbA1c 5.7–6.49)	209 (24.2)
Diabetic (HbA1c > 6.5)	183 (21.2)
	Mean ± SD
Age (years)	48.19 ± 16.44
Weight (kg)	75.87 ± 19.57
Height (cm)	1.58 ± 0.09
BMI (kg/m^2^)	30.21 ± 7.20
Vitamin D (nmol/l)	37.44 ± 23.63
HbA1c (%)	6.01 ± 1.30
FPG (nmol/l)	5.93 ± 1.66
Calcium (nmol/l)	2.29 ± 0.26
TC (nmol/l)	4.73 ± 1.04

BMI = body mass index, DM = Diabetes mellitus, FPG = fasting plasma glucose; HbA1c, glycosylated hemoglobin, SD = standard deviation, TC = total cholesterol.

**Figure 1.**
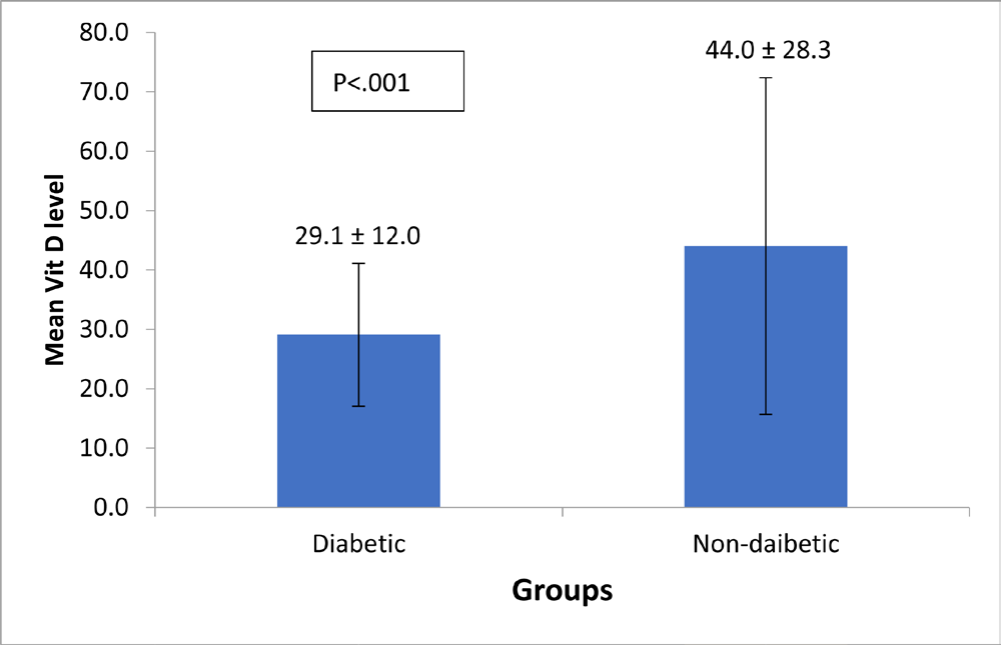
Comparison of vitamin D levels (nmol/L) between diabetic and nondiabetic subjects.

There were significantly lower levels of vitamin D found in diabetic subjects compared to nondiabetic (29.1 ± 12.0 vs 44.0 ± 28.3, *P* < .001; Fig. 1). In comparing baseline characteristics according to vitamin D levels, 368/407 (90.4%) subjects aged > 50 years were vitamin D deficient. A total of 207/209 (99%) subjects with impaired HbA1c and 179/183 (97.8%) people with diabetes had VDD. Interestingly, none of the subjects in the diabetes and impaired HbA1c groups had normal vitamin D levels (Table 2).

**Table 2:** Comparison of basic characteristics according to vitamin D status.

Variables	Vit. D Normal (76–250 nmol/L) 77 (8.9%)	Vit. D Insufficient (50–75 nmol/L) 64 (7.4%)	Vit. D Deficient (<50 nmol/L) 723 (83.7%)	*P* value
Age				
<50 yr	60 (13.1%)	42 (9.2%)	355 (77.7%)	<.001*
>50 yr	17 (4.2%)	22 (5.4%)	368 (90.4%)	
Gender				
Female	61 (8.8%)	54 (7.8%)	575 (83.3%)	.644
Male	16 (9.2%)	10 (5.7%)	148 (85.1%)	
Smoking				
Yes	2 (4.4%)	4 (8.9%)	39 (86.7%)	.603
No	75 (9.2%)	60 (7.3%)	684 (83.5%)	
Education				
College or above	38 (12.3%)	33 (10.7%)	237 (76.9%)	<.001*
High school	13 (12.6%)	7 (6.8%)	83 (80.6%)	
Primary school	26 (5.7%)	24 (5.3%)	403 (89.0%)	
Nationality				
Saudi	53 (10.6%)	44 (8.8%)	404 (80.6%)	.018*
Non-Saudi	24 (6.6%)	20 (5.5%)	319 (87.9%)	
Family history of DM				
Yes	15 (10.4%)	13 (9.0%)	116 (80.6%)	.534
No	62 (8.6%)	51 (71.0%)	607 (84.3%)	
BMI				
Underweight	7 (21.9%)	4 (12.5%)	21 (65.6%)	<.001*
Normal	37 (17.6%)	17 (8.1%)	156 (74.3%)	
Overweight	20 (8.2%)	19 (7.8%)	204 (84.0%)	
Obese	13 (3.4%)	24 (6.3%)	342 (90.2%)	
HbA1C				
Normal (Control; HbA1c < 5.7)	77 (16.3%)	58 (12.3%)	337 (71.4%)	<.001*
Impaired HbA1c 5.7–6.49	0 (0.0%)	2 (1.0%)	207 (99.0%)	
Diabetic HbA1c > 6.5	0 (0.0%)	4 (2.2%)	179 (97.8%)	

* *P* value < .05 was taken significant. Vit D, Vitamin D; BMI, body mass index; DM, diabetes mellitus; HbA1c, glycosylated hemoglobin.

A significant relationship was observed between vitamin D levels and FPG (*P* = .004), calcium (*P* < .013), and total cholesterol (TC) (*P* < .001) among nondiabetic subjects. Among people with diabetes, a substantial correlation of vitamin D was observed with body weight (*P* = .02) and BMI (*P* = .005) (Table 3).

**Table 3:** Correlation of vitamin D with basic characteristics among 3 groups.

Variables	Normal (control) HbA1c (HbA1c < 5.7) r (*P* value)		Impaired HbA1c (HbA1c 5.7–6.49) r (*P* value)		Diabetic (HbA1c > 6.5) r (*P* value)	
	r	*P* value	r	*P* value	r	*P* value
Age (yr)	0.034	.495	0.079	.256	0.084	.260
Weight (kg)	0.017	.732	−0.019	.782	−0.173	.020*
Height (cm)	−0.012	.806	0.001	.998	0.032	.670
BMI (kg/m^2^)	0.026	.610	−0.023	.740	−0.206	.005*
HbA1c (%)	−0.026	.605	0.030	.669	−0.091	.221
FPG (nmol/L)	−0.143	.004	0.106	.126	−0.087	.243
Calcium (nmol/L)	0.124	.013	0.028	.688	0.073	.325
TC (nmol/L)	−0.207	<.001	−0.129	.062	0.039	.597

* *P* value < .05 was taken significant. BMI, body mass index; FPG, fasting plasma glucose; HbA1c, glycosylated hemoglobin; TC, total cholesterol.

The univariate and multivariate logistic regression analyses showed a significant association between VDD and being > 50 years of age, overweight, or obese (Table 4). VDD was associated with age, HbA1c, FPG, calcium, and TC (Table 5).

**Table 4:** Logistic regression analysis of different variables and Vitamin D deficiency.

Variables	Univariate analysis		Multivariate analysis	
	OR (CI 95%)	*P* value	OR (CI 95%)	*P* value
Age				
<50 yr	1		1	
>50 yr	3.5 (1.99–6.05)	<.001*	2.23 (1.20–4.12)	.011*
Gender				
Female	1			
Male	0.96 (0.54–1.71)	.883	0.82 (0.44–1.53)	.526
Smoking				
No	1			
Yes	2.17 (0.52–9.13)	.292	2.44 (0.52–11.5)	.261
Education				
Primary school	1		1	
High school	0.42 (0.21–0.85)	.016*	0.50 (0.23–1.01)	.085
College or above	0.43 (0.26–0.73)	.002*	0.77 (0.43–1.39)	.390
Nationality				
Saudi	1		1	
Non-Saudi	1.67 (1.01–2.76)	.045*	1.31 (0.77–2.24)	.316
Family history of DM				
No	1		1	
Yes	0.81 (0.45–1.47)	.488	0.79 (0.41–1.54)	.495
BMI				
Normal weight	1		1	
Underweight	0.76 (0.31–1.90)	.562	0.94 (0.36–2.45)	.902
Overweight	2.38 (1.34–4.26)	.003*	2.15 (1.19–3.88)	.011*
Obese	6.02 (3.12–11.6)	<.001*	4.93 (2.49–9.74)	<.001*

* *P*-value < .05 was taken significant. BMI, body mass index; DM, diabetes mellitus.

**Table 5:** Logistic regression analysis of different variables and vitamin D deficiency.

Variables	Univariate analysis		Multivariate analysis	
	OR (CI 95%)	*P* value	OR (CI 95%)	*P* value
Age (years)	1.07 (1.05–1.09)	<.001*	1.04 (1.02–1.06)	<.001*
Weight (kg)	1.04 (1.02–1.05)	<.001*	1.10 (0.96–1.26)	.166
Height (cm)	1.19 (0.09–16.4)	.897	0.001 (0.00–71.6)	.211
BMI (kg/m^2^)	1.10 (1.06–1.14)	<.001*	0.82 (0.59–1.13)	.224
HbA1c (%)	4.65 (2.83–7.64)	<.001*	2.71 (1.46–5.00)	.002*
FPG (nmol/l)	5.43 (3.32–8.86)	<.001*	3.52 (1.97–6.28)	<.001*
Calcium (nmol/l)	0.33 (0.17–0.63)	.001*	0.28 (0.13–0.59)	.001*
TC (nmol/l)	1.86 (1.43–2.41)	<.001*	1.65 (1.23–2.21)	.001*

* *P* value < .05 was taken significant. BMI, body mass index; FPG, fasting plasma glucose; HbA1c, glycosylated hemoglobin; TC, total cholesterol.

## 4. Discussion

In the present study, VDD was common, with an incidence of 83.7%. A significantly lower level of vitamin D was found in diabetic subjects compared to nondiabetic subjects (*P* < .001), and there were no significant differences found between the genders according to vitamin D status. While none of the subjects in diabetes and impaired HbA1c groups had normal vitamin D levels, a significant association was found between VDD and those older than 50, overweight, or obese, as well as with HbA1c, FPG, calcium, and TC levels.

A study reported a 91.4% incidence rate of VDD among subjects with T2DM and reported significantly lower levels of vitamin D among those subjects compared to the control group. They found no significant difference between VDD and sufficient control subjects.^[16]^ Another study stated that 72% of type 2 diabetics were found to have VDD, which was associated with poor glycemic control. Inverse relationships of vitamin D levels with FPG and postprandial glucose levels were observed. Similar to the present study, an inverse relationship between low vitamin D and elevations in HbA1c was reported.^[17]^ Recent Indian and Chinese studies have reported 75% and 64% of VDD among T2DM individuals, respectively.^[18,19]^

A Saudi study reported a 47.3% incidence of VDD among T2DM patients. Likewise, a significant difference was observed between VDD and higher HbA1c levels compared to non-vitamin D deficient diabetics with normal HbA1c (*P* < .001). Similar to the current study, they reported a significant association between VDD and several risk factors, including age, gender, and HbA1c among people with diabetes.^[20]^ In contrast to the present study, they also reported a significant association between VDD and gender among people with diabetes.^[20]^

Our study found 97.8% of the diabetic subjects had VDD and significantly lower levels of vitamin D. Another study reported 64.3% of subjects with VDD were among people with diabetes; however, VDD did not significantly affect correlations with BMI, weight, or height, as opposed to the present study.^[21]^ In contrast to the results of present study, a study found no statistically significant difference in the mean values of vitamin D among diabetics and nondiabetics. They also reported no significant association between vitamin D levels and HbA1c.^[22]^ In both studies, the sample sizes were small (50 and 75, respectively), and most patients were obese. Thus, bias in their results would have been inevitable. The present study findings are different, possibly because of the small sample sizes and biased selections of study subjects in the prior studies.

An Australian study showed a significant inverse relationship between VDD and age, gender, BMI, nationality, smoking, and lipid profile among people with diabetes.^[23]^ Wang et al, 2023 reported that women with T2DM exhibited increased susceptibility to VDD, and there was an inverse correlation between vitamin D levels and HbA1c.^[19]^ In a Brazilian study, the prevalence of hypovitaminosis D in T2DM was correlated with a high BMI, obesity, and HbA1c.^[24]^ Similarly, a German study reported that VDD was linked with the prevalence of T2DM and severe VDD was significantly associated with increasing FPG levels and HbA1c.^[25]^ In a Korean study, a significant association of VDD with increasing HbA1c levels was found; however, no such significance was reported between vitamin D and FPG levels among T2DM patients.^[26]^ Likewise, due to cultural and geographical differences, a difference in the significance of a relationship of vitamin D with HbA1c and FPG might have been observed. Based on the studies discussed above, VDD is a key component in the developing or poor prognosis of T2DM. The body of literature indicates that the advantage of administering vitamin D for reducing diabetes risk stems from vitamin D effects on the action of insulin.^[27]^ Vitamin D receptor expression in beta cells of the pancreas highlights the importance of vitamin D in the functioning of beta cells and insulin secretion. In VDD, the functioning of beta cells may be compromised, leading to diminished insulin secretion.^[28]^ Indirectly, vitamin D might also influence the action of insulin through a calcium-mediated effect. Because vitamin D tightly regulates calcium homeostasis, intracellular calcium levels are needed to ensure effective insulin action in different body parts.^[29]^ In addition, vitamin D regulates the renin-angiotensin system, endothelial vasodilation, and levels of lipids, and a high level of vitamin D is associated with low FPG and HbA1c levels.

The present results showed that the incidence of VDD was widespread among study subjects with diabetes, and its deficiency was evident in the impaired diabetics as well. Moreover, a significant association between VDD and HbA1c and FPG indicates the importance of measurement of vitamin D in type 2 DM patients. It is suggested that VDD could be used as an early sign of DM development. In this regard, some longitudinal studies need to be carried out to confirm the present study assumption.

The current study might not be free from limitations. First, the study was restricted to a single center; hence, subjects might not represent the total population. Second, the present study was retrospective, using data gathered from hospital records; therefore, selection bias and observer bias could not be completely ruled out. Use of vitamin D supplements was not recorded from the data available, nor was exposure to sunlight measured, and the season of vitamin D collection was not mentioned. Therefore, potential associations of vitamin D could not be determined with complete accuracy.

## 5. Conclusions

The present study found a high incidence of VDD and a significantly lower level of vitamin D in people with diabetes, and age, being overweight, obesity, HbA1c, and FPG were the few determinants of VDD among T2DM patients. Further research is needed to confirm that the VDD could be a valuable early sign of developing T2DM.

## Acknowledgments

This work was funded by the Deanship of Scientific Research (DSR), KAU, Jeddah, Saudi Arabia, under grant No. DF-345-140-1441. The authors acknowledge DSR with thanks for their technical and financial support. The funders had no role in study design, data collection and analysis, decision to publish, or preparation of the manuscript.

## Author contributions

**Conceptualization:** Sami Hamdan Alzahrani, Khaled A. Yaghmour, Sarah Al Muammar.

**Data curation:** Khaled A. Yaghmour.

**Formal analysis:** Mukhtiar Baig.

**Funding acquisition:** Sami Hamdan Alzahrani.

**Investigation:** Mukhtiar Baig, Khaled A. Yaghmour, Sarah Al Muammar.

**Methodology:** Sami Hamdan Alzahrani, Mukhtiar Baig, Khaled A. Yaghmour, Sarah Al Muammar.

**Project administration:** Sami Hamdan Alzahrani, Mukhtiar Baig, Sarah Al Muammar.

**Writing – original draft:** Khaled A. Yaghmour, Sarah Al Muammar.

**Writing – review & editing:** Sami Hamdan Alzahrani, Mukhtiar Baig.
